# Replication study: the case of disappearing teaspoons in a Scottish neuroscience department

**DOI:** 10.1093/braincomms/fcag064

**Published:** 2026-04-01

**Authors:** Tara L Spires-Jones, Jane Tulloch

**Affiliations:** The Centre for Discovery Brain Sciences and UK Dementia Research Institute at the University of Edinburgh, Scotland EH8 9JZ, UK; The Centre for Discovery Brain Sciences and UK Dementia Research Institute at the University of Edinburgh, Scotland EH8 9JZ, UK

**Keywords:** teaspoon displacement, spoon theft, neuroscience, spoon pilfering

## Abstract

In the landmark paper by Lim, Hellard, and Aitken published in 2005, the rate of loss of workplace teaspoons was determined for the Macfarlane Burnet Institute for Medical Research and Public Health in Melbourne, Australia. In this institute of 140 people, the half-life of teaspoons in communal tearooms was 42 days and the rate of loss was not influenced by the teaspoons’ value. Authors concluded that the loss of workplace teaspoons was rapid, influencing both teaspoon availability and office culture more generally. Over 20 years later, the issue of teaspoon loss from common rooms has not abated, leaving the question ‘where have all the bloody teaspoons gone?’ unanswered. In this study, we replicated the experiment of Lim and colleagues in a Scottish neuroscience department. 48 teaspoons (24 gold-coloured and 24 much cheaper standard silver coloured) were purchased and placed in the common room of our building which houses 82 researchers. Spoons were counted weekly to monitor attrition. We observed gold teaspoons started disappearing within the first week of data collection and over the course of 10 months of observation, over 66% of all spoons disappeared. The half-life of teaspoons was 182 days for gold spoons and 280 days for silver spoons. A linear model showed significant effects of both time and spoon type. In conclusion, we replicated Lim and colleagues data showing teaspoons are stolen from academic department common rooms, however our data from Scotland show that the more expensive teaspoons were stolen more than the cheaper spoons, contrary to the findings in Melbourne. Pilfering of teaspoons from academic common room remains a problem that warrants further research as it impacts the wellbeing of scientists, at least in two institutes on opposite sides of the globe.

## Teaser

Spires-Jones and Tulloch conducted an experiment studying teaspoon pilfering from their departmental common room. They observed 66% of teaspoons disappeared within 10-months with gold teaspoons disappearing faster than standard spoons. These data indicate that spoon displacement is an ongoing issue in academia that may affect the wellbeing of translational neuroscientists.

## Graphical Abstract

**Graphical Abstract:**
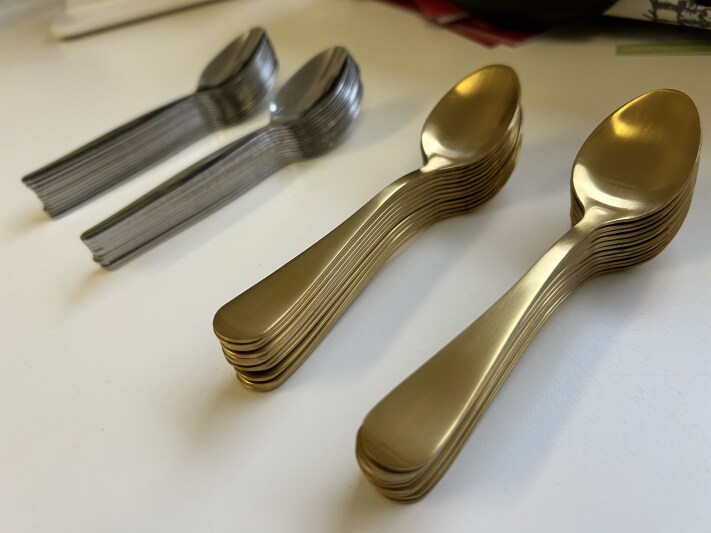

## Introduction

The cutlery bin in the common room of the Neuroscience building in the Centre for Discovery Brain Sciences at the University of Edinburgh has been the topic of lunchtime conversation for many years. Most of these conversations centre on disappearing cutlery (forks and spoons in particular) and the annoyance this causes. In one common room lunch, author TS-J pointed out the landmark paper by Lim *et al*.^1^ in which epidemiologists in Melbourne had quantified a similar phenomenon.^1^ This seminal work quickly became the lab members’ favourite paper and a copy was printed and taped over the cupboard containing the cutlery bin to try and inspire people to steal less cutlery. The inspiration failed and cutlery disappearance continued to be a point of contention in the department. Based on this issue and the desire to contribute to rigorous, robust scientific conversation, we decided to replicate Lim *et al.*’s^1^ study.

In this study, we tested the following hypotheses:

Hypothesis 1: Scientists working in our institute will steal teaspoons at a similar rate to those in Lim *et al.*^1^

Null hypothesis 1: Scientists here are angels who will not steal spoons.

Hypothesis 2: The rate of stealing gold spoons will differ from that of normal silver spoons (note, JT thought gold would go faster TSJ thought they would go slower as more identifiable as common room spoons).

See null hypothesis 1.

## Materials and methods

**Figure 1:**
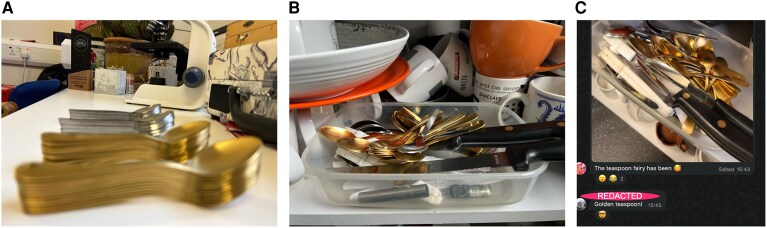
**Standard and gold teaspoons before the experiment began** (**A**). Experimental teaspoons were sneakily delivered to the common room cutlery bin at 10:15 a.m. 13 February 2025 (**B**). Members of the department notice the teaspoons and the lab WhatsApp chat explodes from 15:43 on February 13th (**C**). The gold teaspoons are 5.56 inches (14.1 cm) long for scale.

Author TS-J purchased 24 gold teaspoons (£16.99) and 24 standard teaspoons (£7.18) from an online retailer on 10th February 2025. Note these were not purchased with lab or department funds as there is a dearth of grant funding for teaspoon related research. Spoons were transported from TS-J’s home to the laboratory building and photographed (Fig. 1A). At 10:15 a.m. on 13 February 2025, all 48 spoons were dumped into the common room cutlery bin in a cupboard (Fig. 1B). Author JT furtively counted spoons weekly on a Thursday morning except for weeks when she was on leave. Data were recorded in a spreadsheet saved in a secret location on the lab server to avoid any lab members becoming aware of the nature of the experiment. Data were analysed and plotted in R. A linear model was used to assess effects of time and spoon type on the number of spoons [lm_spoons < - lm(n_spoons ∼ spoon_type × date, data = SpoonData)]. Results were presented in a lab meeting in January 2026. Ethical approval for the study was granted *post-hoc* via WhatsApp committee.

## Results

Approximately 5.5 h after spoons were placed in the common room, lab member JC commented in the group WhatsApp chat ‘The teaspoon fairy has been’ with a photo of said spoons. There were subsequent mind blown emojis and exclamations about the golden teaspoons specifically from lab member FG (Fig. 1C). More speculation followed about the mystery teaspoon gifter. Authors JT and TS-J spent most of the rest of the day giggling.

**Figure 2:**
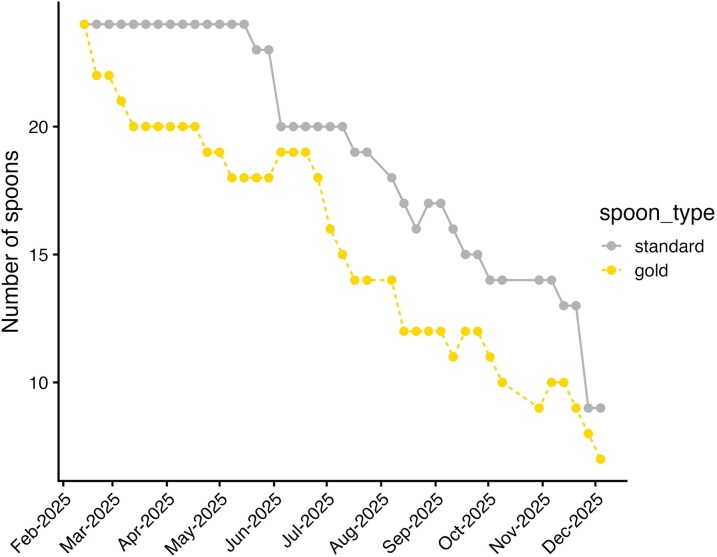
**Quantification of teaspoon numbers from February to December 2025 shows a steep drop in ‘gold beauties’ from the start. ANOVA after a linear model shows a significant effect of time (*F*[1,76] = 1242, *P* < 2e−16) and of spoon type (*F*[1,76] = 216, *P* < 2e−16). At the start of the experiment *N* = 24 of each spoon type.** The experimental unit was an individual spoon.

Counts of spoon number (Fig. 2) revealed that gold teaspoons began to disappear within the first week of data collection. All of the original 24 standard spoons remained until 22 May 2025; 3 months after data collection began. Statistical analysis confirms that there was a significant effect of both time (*F*[1,76] = 1242, *P* < 2e−16) and of spoon type (*F*[1,76] = 216, *P* < 2e−16) on the number of spoons remaining. There was not a significant interaction between spoon type and time. We calculate that the half-life of gold spoons was 182 days and standard spoon half-life was 280 days. These data partially support our two hypotheses. We did observe that scientists in our institute steal teaspoons, however this was at a slower rate than observed in Lim *et al*.’s^1^ study where they observed a half-life of 42 days for teaspoons in communal rooms. We further observed a difference between disappearance of gold and standard spoons with the more expensive spoons being pilfered faster.

After revealing study results at the lab meeting, there was minimal admission of spoon thievery with most people claiming they ‘borrowed’ spoons but returned them.

## Discussion

Teaspoons are an essential component of any research institute. Discussions after this experiment reveal that some people use a spoon daily to eat their ‘mousse’ while many others use them for dispensing instant coffee, fishing tea bags out of cups, or adding sugar or milk and stirring their beverage of choice. Many academics in our institute acknowledge the central role of caffeinated beverages in our workday. The chronic lack of teaspoons and other cutlery negatively impacts on the wellbeing of researchers. From this study, we conclude that in our building, people indeed steal teaspoons from the common room with gold teaspoons being more commonly stolen. We also conclude that author JT, our lab manager, is always right. She correctly predicted that gold teaspoons would be stolen more while TSJ naively thought that these would be too identifiable as belonging in the common room so would be stolen less.

Where the stolen teaspoons have gone remains a mystery. We did observe a few appearing in places outside of the common room in the building, but most remain untraceable. Lim *et al*.^1^ proposed based on work from Douglas Adams that ‘Somewhere in the cosmos, along with all the planets inhabited by humanoids, reptiloids, walking treeoids, and super intelligent shades of the colour blue, a planet is entirely given over to spoon life-forms. Unattended spoons make their way to this planet, slipping away through space to a world where they enjoy a uniquely spoonoid lifestyle, responding to highly spoon oriented stimuli, and generally leading the spoon equivalent of the good life.’^2^ While we have no direct evidence to support this theory, the authors’ anecdotal experience as mothers of teenage boys does lead us to suspect that both spoons and individual socks from pairs can indeed disappear through portals to other worlds.

As pointed out by an esteemed peer reviewer, one limitation of our study was the lack of accounting for possible contamination of third-source silver teaspoons that were present in the common room at the beginning of this experiment. We grudgingly acknowledge that this could have contributed to the differences in half-life between spoon types. The reviewer also raised the point that regional preferences for tea versus coffee could affect the experimental measurements. According to random Australians on Reddit who were asked this question, Australians like both tea and coffee equally with one person stating ‘inject all the hot drinks into my eyeballs plx.’^3^ The wisdom of Redditers on Scottish hot beverage habits leans towards tea as more popular with comments such as ‘I was drinking tea earlier but now I’m drinking red wine, from a tea cup.’^4^ We argue that whether tea or coffee is the hot beverage of choice does not influence the need for teaspoons as much as whether people add sugar or other stirrable additives to the beverages. Nor does this account for other spoon-related habits like eating yogurts, soup, etc.

Future work that was suggested in our lab meeting discussion of this study included examining migration of other types of cutlery, particularly forks. This is another key problem as eating salad for lunch with a spoon proves tricky, and several rounds of replacement forks have not solved our common room shortage.

In conclusion, we encourage all research institutes to consider the implications of common room teaspoon disappearance for research productivity and scientist wellbeing.

## Data Availability

All data in the study are included in the figures. The spreadsheet including comments about how much we were laughing is available upon request to the corresponding author.
